# Clinical Efficacy and Tolerability of Lemon Balm (*Melissa officinalis* L.) in Psychological Well-Being: A Review

**DOI:** 10.3390/nu16203545

**Published:** 2024-10-18

**Authors:** Imogen Maria Mathews, Jessica Eastwood, Daniel Joseph Lamport, Romain Le Cozannet, Pascale Fanca-Berthon, Claire Michelle Williams

**Affiliations:** 1 School of Psychology & Clinical Language Sciences, University of Reading, Earley Gate, Whiteknights Road, Reading RG6 6ES, UK; i.m.mathews@pgr.reading.ac.uk (I.M.M.); jessica.eastwood@reading.ac.uk (J.E.); daniel.lamport@reading.ac.uk (D.J.L.); 2 Givaudan France Naturals, 250 rue Pierre Bayle, BP 81218, 84911 Avignon, France; romain.le_cozannet@givaudan.com (R.L.C.); pascale.fanca-berthon@givaudan.com (P.F.-B.)

**Keywords:** polyphenols, triterpenoids, essential oils, anti-depressant, anxiolytic, cognition, sleep

## Abstract

Background: There is renewed interest in the use of ancient herbal remedies for their potential health benefits, particularly in the psychological domain. One herb that is receiving growing attention is lemon balm (*Melissa officinalis* L.) which has received considerable interest for its influence on the brain. Lemon balm boasts an array of phytochemicals, including rosmarinic acid, citral, oleanolic acid, and ursolic acid, which are believed to underpin these effects on psychological well-being. Pharmacological evidence from animal and cellular work reveals that lemon balm and its components may modulate several brain signalling pathways, including GABAergic, cholinergic, and serotonergic systems. Results/Conclusions: Although further robust randomised controlled trials using lemon balm are required, existing research indicates that lemon balm holds promise as a calming agent exhibiting both anxiolytic and anti-depressant properties and can elicit cognitive and sleep-quality enhancement.

## 1. Introduction

Lemon balm is a perennial herbaceous plant renowned for its aromatic leaves and numerous medicinal properties [1]. Belonging to the mint family (Lamiaceae), lemon balm is native to the Mediterranean region but is now cultivated and naturalized in various parts of the world, including Southern Europe, North Africa, and Western Asia [2]. Lemon balm has a 2000-year history, appearing in records of the Historica Plantarum in approximately 300 B.C. and in Dioscorides’ “De Materia Medica” in approximately 50–80 B.C., where its medicinal properties are described [1,3,4]. In herbal medicine systems, the use of lemon balm has been well documented, and it has been traditionally employed to alleviate digestive issues, promote relaxation and sleep, improve mood, soothe skin irritations, and promote wound healing. In more modern times, lemon balm continues to be utilised for medicinal benefits, with documented use in various pharmacopoeias such as the British Herbal Pharmacopoeia [5], the European Pharmacopoeia [6], and the Iranian Herbal Pharmacopoeia [7] for treatment of anxiety and stress management, treatment of sleep disorders, cognitive enhancement, antiviral activity, and digestive health [8]. Overall, lemon balm in various forms, including teas, tinctures, essential oils, and dietary supplements, occupies a prominent place in the realm of herbal medicine and complementary therapies, offering a natural approach to enhancing health and well-being. However, few robust clinical trials have been published thus far. This narrative review will critically evaluate the available evidence documenting the potential beneficial effects of lemon balm on psychological well-being, sleep, and cognitive function across the lifespan. Additionally, we will describe the proposed active ingredients of lemon balm and review the available pharmacological and pharmacokinetic evidence to explore which mechanisms of action may be of importance for these psychological effects.

## 2. Results

### 2.1. Key Chemical Components of Lemon Balm

**Figure 1:**
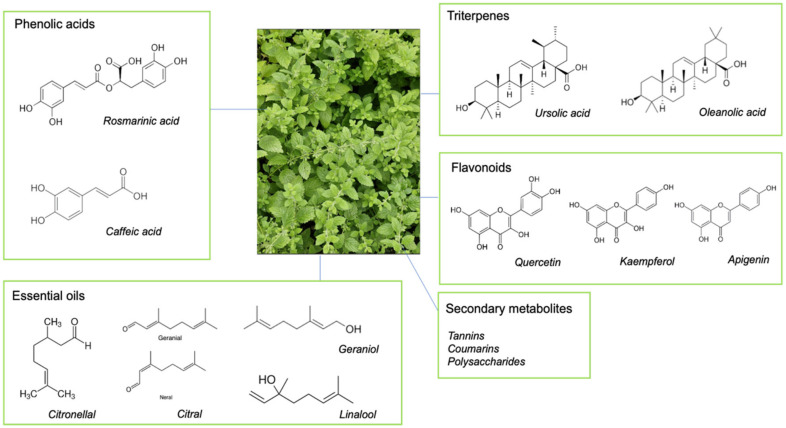
Chemical structures of the main active components present in *Melissa Officinalis* L. (lemon balm) herb, including phenolic acids [15], triterpenes [16], flavonoids [12,17], essential oils [18], and secondary metabolites [19].

The phytochemistry of lemon balm includes a variety of compounds such as essential oils, phenolic acids, flavonoids, triterpenes and other secondary metabolites such as tannins, coumarins, and polysaccharides. An exhaustive breakdown of all phytochemical components present in lemon balm can be found in a recent review [9], while key components for which there is mechanistic evidence are outlined here in Figure 1. The composition of the essential oils can vary depending on several factors, such as the plant’s growing conditions and the method for their extraction. However, key components include citral, citronellal, geraniol, and linalool oils [10]. The phenolic acids, composed primarily of rosmarinic acid (RA) and caffeic acid, are significant for their antioxidant properties, whilst the flavonoids in lemon balm (such as quercetin, kaempferol, and apigenin) contribute to its antioxidant, anti-inflammatory, and neuroprotective properties [11,12]. Ursolic acid and oleanolic acid are the major triterpenes present in lemon balm, and each has well-documented anti-inflammatory and anti-microbial properties [13]. Despite lemon balm comprising a plethora of phytochemicals, RA has become the main biomarker for plant standardisation and quality control given its high concentration relative to the other components found in the plant [14].

### 2.2. Biological Mechanisms of Action of Lemon Balm

Researchers have identified a host of complex interactions through which lemon balm and its metabolites may operate to produce beneficial effects on psychological well-being, sleep, and cognition. The majority of these actions occur directly in the brain, modulating several neurochemical pathways and signalling molecules, including GABAergic and cholinergic pathways. Several indirect actions, for example, modulating the HPA axis, have additionally been described. More recently, there is also growing evidence highlighting the importance of gut–brain interactions in mediating the beneficial effects of lemon balm. Whilst the potential mechanisms of action outlined below are not exhaustive, they represent a brief summary of the major routes for the biological action of lemon balm.

- GABAergic activity: Lemon balm influences GABAergic activity through various mechanisms, primarily by enhancing the activity of GABA, an inhibitory neurotransmitter in the central nervous system [11,12]. Rosmarinic acid has been shown to inhibit GABA transaminase, the enzyme responsible for the degradation of GABA, leading to the accumulation of GABA levels in the brain [1,20]. Numerous compounds in lemon balm, including RA, can directly bind to GABA receptors such as GABA_A_ [21], enhancing their inhibitory action [22,23]. This agonist activity at GABA_A_ receptors by RA shares similar mechanisms to approved pharmacological therapies for insomnia such as benzodiazepines [12,24].
- Cholinergic modulation: Lemon balm can also influence cholinergic activity; both RA [25] and terpenoids [26] can inhibit acetylcholinesterase (AChE)—a key therapeutic target for Alzheimer’s management [27]. AChE is the enzyme responsible for breaking down acetylcholine—leading to increased levels of acetylcholine in the brain and enhanced cholinergic transmission. Other, as yet unidentified, compounds in lemon balm have been found to interact directly with nicotinic and muscarinic acetylcholine receptors [28], but it is still unclear how lemon balm or its components interact with subtypes of muscarinic (including M1, M2, and M4 receptors) and nicotinic (including α4β2 and α7 subtypes) receptors [12,29].
- Antioxidant activity: Lemon balm exhibits significant antioxidant activity due to its rich content of phenolic compounds, flavonoids, and essential oils. RA, caffeic acid, and flavonoids are potent antioxidants that help neutralize free radicals, thereby reducing oxidative stress and protecting cells from damage [25,30]. Additionally, there is evidence that lemon balm can inhibit lipid peroxidation [31], chelate metal ions [32], and modulate the activity of antioxidant enzymes [33], making it a valuable herb for protecting against oxidative stress and related cellular damage.
- Anti-inflammatory effects: Lemon balm has demonstrated significant anti-inflammatory activity, attributed to its rich content of phenolic acids, flavonoids, and essential oils. These compounds act through various mechanisms to reduce inflammation. Firstly, lemon balm can inhibit the production of pro-inflammatory cytokines, which play a crucial role in the inflammatory response [34]. Lemon balm also contains compounds that inhibit cyclooxygenase (COX) and lipoxygenase (LOX), which are enzymes involved in the production of inflammatory mediators such as prostaglandins and leukotriennes [35]. Lemon balm can inhibit the NF-κB signalling pathway, a key regulator of inflammation [36]. Finally, lemon balm can modulate the activity of various enzymes involved in inflammation [22].
- Gut microbiota: Lemon balm has been shown to influence the gut microbiota, primarily through its antimicrobial, anti-inflammatory, and antioxidant properties. Extracts of lemon balm exhibited antimicrobial activity against several strains of bacteria, suggesting a role in modulating gut microbiota by inhibiting harmful pathogens, thereby promoting a healthier microbial balance [25]. Some studies suggest that lemon balm may have prebiotic effects promoting the growth of beneficial gut bacteria, such as *Bifidobacterium animalis* [37]. The anti-inflammatory and antioxidant properties of lemon balm (outlined above) can also help reduce inflammation and protect gut cells from oxidative stress, creating a more favourable environment for beneficial bacteria to thrive [35,36]. Finally, lemon balm has been shown to modulate the overall composition of gut microbiota, promoting a balance that supports health [38]. Together, these effects contribute to a healthier gut environment, supporting the growth of beneficial bacteria and inhibiting pathogenic bacteria.

### 2.3. Psychological Outcomes Following Lemon Balm Supplementation

#### 2.3.1. Infants and Children

Table 1 outlines the main study design characteristics and findings from lemon balm studies on infants and children. Infants with colicky symptoms responded with reduced crying time after taking a daily formulation containing 130 mg of lemon balm for 28 days [39]. Similarly, colicky infants also showed a daily significant reduction in crying time when using a formulation containing 38.75 mg/kg/day lemon balm administered for 7 days [40]. Both studies associated the reduction in crying time with lemon balm’s calming properties and anti-anxiety effects.

Primary school children experiencing sleep and/or cognitive problems have also been supplemented with lemon balm and valerian combinations with a view to reducing the associated symptoms of poor concentration and sleep. Specifically, lemon balm capsules of up to 320 mg and valerian tablets of up to 640 mg taken together daily for 28 days were associated with significant improvements in sleep-quality and restlessness scores relative to baseline [41]. Similarly, significant improvements in the ability to concentrate and reduced distractibility relative to baseline were seen after a 7-week intervention with a daily dose of 320 mg lemon balm and 640 mg valerian [42].

Lemon balm has also been shown to reduce pre-menstrual symptoms in adolescent females aged 14–18. Supplementing 1200 mg lemon balm daily over three consecutive menstrual cycles significantly reduced anxiety, depression, and insomnia scores compared to PMS management workshops or a starch-based placebo in 200 adolescent females up to the age of 18 [43]. In a follow-up study where PMS management workshops were removed to solely see the effects of lemon balm, Heydari and colleagues found significant decreases in insomnia, anxiety, depression, and social dysfunction scores following 1200 mg lemon balm relative to placebo [44].

In summary, lemon balm trials involving infants and children have shown anti-anxiety, cognitive, and sleep-enhancing effects in open-label studies, with good tolerance. However, methodological variability such as an absence of fixed doses between studies makes it difficult to compare study efficacy and tolerability.

**Table 1:** Key characteristics of included experimental trials using lemon balm and botanical combinations in infants and children.

Citation	Design	Participants			Intervention		Design Method	Findings
		No.	Age	Population	Duration	Dosage (E: Experimental Group, C: Control Group)		
Akbarzadeh et al. (2018) [43]	RCT	200	14–18	Adolescent females experiencing PMS	3 consecutive months (d per respective menstrual cycle treatment)	E1: 2 × 0.6g lemon balm capsules/d per cycle (n = 50) E2: CEP per cycle (n = 50) C1: 2 × 0.6 g starch-based placebo capsules/d per cycle and CEP (n = 50) C2: education programme without training (n = 50)	*Baseline measurements:* Well-being affected by PMS entry (PSST > 20 and GHQ < 23) w 0, 4, 8 & 16: *Wellbeing assessment:* PSST (emotional, social, and physical specific to PMS) *Tolerability:* Assessed verbal reports of AEs at w16	Group E1 (at w 16 compared to Groups E2, C1, and C2): ↓ Overall emotional, social, and physical symptoms to E2, C1, and C2 C2 effective to C2, and C1 showing that lemon balm, and CEP can be effective in PMS sufferers No AEs developed Dropouts (n = 0)
Gromball et al. (2014) [42]	Prospective multi-centre non-interventional study	169	6–11	Children with hyperactivity, concentration, and impulsiveness problems (not meeting ADHD in DSM-IV criteria)	7 weeks	E: average 2 × 0.16 g lemon balm and 0.32 g valerian capsules/d (n = 169). No placebo	*Baseline assessment:* No ADHD criteria, but existing sleep and concentration problems (parent and paediatrician Obs). w 0, 2, and 7: *Concentration and sleep-quality assessments:* Researcher visits and parents’ continuous home behaviour Obs scored 0–5 (5 = severe). *Tolerability:* AEs reported by parents	Group E (at w 7 compared to w 0): Paediatricians Obs ↑ concentration, ↓ impulsivity, difficulty falling asleep, and morning fatigue Parents reported ↓ distractibility No significant differences emerged between school and family environments from parents reports of concentration difficulties No significant AEs reported Dropouts (n = 93) based on participant or paediatrician decision
Heydari et al. (2019) [44]	Double-blind RCT	100	14–18	Adolescent females experiencing PMS	3 consecutive months (d per respective menstrual cycle treatment)	E: 2 × 0.6 g lemon balm capsules/d (n = 50) C: 2 × 0.6 g starch-based placebo capsules/d (n = 50)	*Baseline assessment: * Well-being affected by PMS entry (PSST > 20) W 0 and 16: *Well-being assessment*: GHQ-28 *Tolerability:* AEs verbally reported at w 16	Group E (compared to Group C at w 16): ↓ Psychosomatic problems ↓ Anxiety and sleep disturbance ↓ Social function problems ↓ Psychosomatic problems No significant AEs reported Dropouts (n = 0)
Martinelli et al. (2017) [39]	Multi-centre prospective randomised comparative study	176	≥2 weeks to 4 months	Infants with IC	28 days	E1: 1 × 1 mL of 0.13 g lemon balm, 0.018 g chamomile, 2 × 10^9^ tyndallized *Lactobacillus* *acidophilus*/d (n = 60) E2: 1× *Lactobacillus reuteri* (10^8^ CFU) in 5 drops/d (n = 60) C: 2 × 15 drops in 0.06 mg Simethicone/d (n = 60)	*Baseline assessment:* *Anxiety entry scoring:* Frequent and severity of crying episodes using Rome III criteria D 0, 7, 14, 21, and 28: *Anxiety and gastrointestinal* *assessments:* parents filled in maternal diary (crying time frequency and eating habits/stool frequency) Tolerability: assessed AEs throughout	Group E1 (compared to Group E2 and C at d 28): ↓ Crying time (anxiety) to C Homogenous crying time improvement to E2 No AEs reported Dropouts (n = 0)
Müller & Klement (2006) [41]	Open multicentre post-marketing surveillance study	918	*M* = 8.3	Children with restlessness and dyssomnia	*M* = 4-weeks (range 3–5 weeks)	E: variable dose of 3.5–4 × 0.08 g lemon balm and 0.16g valerian capsules/d (n = 918) No placebo	*Baseline assessment:* Restlessness and/or dyssomnia problems >10 months (physician Obs) W 0 and 4: *Sleep and restlessness assessment:* Physician visits and parents’ feedback at visits Symptom severity then weighted on Obs of efficacy and improvement scoring, 0–5 (1 = very good, 5 = poor) *Tolerability:* AEs reported by physicians over whole study phase (1 = very good to 5 = poor)	Group E (at w 4 compared to w 0): ↓ Sleep problems in the 744/918 children with initial dyssomnia ↓ Restlessness in the 646/918 sufferers improved Dropouts (n = 142) not related to “poor tolerability,” for various other reasons No intervention-related AEs reported
Savino et al. (2005) [40]	Double-blind RCT	93	21–60 days	Infants with IC	21 days (7-day intervention with a 14-day follow up)	E: 2 × 1 mL containing 0.01937 g lemon balm, 0.0328 g chamomile, 0.0355 g fennel powdered sachet/d (n = 41) C: 2 × 1 mL matched placebo with only vitamins, no herbals (n = 47)	*Baseline assessment:* *Anxiety entry scoring:* Frequent and severity of crying episodes using Wessel’s criteria (>3 h frequency, >3 wks severity) and 3 d Obs D 0, 7, and 21: *Anxiety and gastrointestinal assessments:* parents filled in structured diary (crying time frequency/severity and medication administration, AE development)	Group E (compared to Group C)): At d 7: crying time (anxiety) ↓ At d 21: crying time (anxiety) ↓ Dropouts (n = 5) but unrelated to trial No AE recorded throughout study period

AE, adverse event; CEP, care educational programme; IC, infantile colic; GHQ, General Health Questionnaire; PMS, pre-menstrual syndrome; PSST, pre-menstrual syndrome screening tool; Obs, observations; ↑, increased; ↓, decreased.

#### 2.3.2. Young Adults

Table 2 summarises the outcomes and study design features from studies in young adults aged 18 to 30. Several studies have assessed acute lemon balm effects on mood and cognitive performance in stress and non-stress conditions in healthy young adults. Kennedy and colleagues conducted two trials assessing the acute effects of lemon balm on cognitive function under non-stress conditions utilising the CDR task battery at baseline and 1, 2.5, 4, and 6 h [45], and at 1, 3, and 6 h [46] following lemon balm intervention. The original study randomised participants to receive single doses of 300 mg, 600 mg, or 900 mg of lemon balm in a cross-over design [45], while the follow-up utilised higher doses of 600 mg, 1000 mg, or 1600 mg in the same manner [46]. Both studies also included a placebo control condition. In the original study (2002), accuracy of attention was improved following only the 600 mg dose, and this improvement persisted at all time points post-intervention. However, word recognition and spatial memory scores were impaired following all three doses in a dose-dependent manner across the follow-up time points. In contrast, the latter study found that memory was improved 3 and 6 h post-intervention following the highest 1600 mg dose of lemon balm compared to the placebo [46]. There were, however, no effects on attention, as seen in the previous study, and speed of processing across attention and memory tasks was impaired following lemon balm administration, particularly at the lower doses, compared to the placebo. Finally, and in complete contrast again, a later study performed under stressful conditions induced by a stress-based task named the Defined Intensity Stressor Stimulation (DISS) [47] found a 300 mg dose of lemon balm to improve the speed and accuracy of mathematical processing post-DISS compared to placebo. As such, findings for acute effects on cognitive function appear mixed, with no clear beneficial effect or optimum dose.

In addition to cognitive function, Kennedy and colleagues assessed the effect of lemon balm on mood, under both non-stress [45,46] and stressful conditions [47]. In the first study, subjects rated their feelings of calmness significantly higher 1 h after consuming the 300 mg and 900 mg doses compared to the placebo, and this increase in calm persisted at the 2.5 timepoint following the 300 mg dose [45]. While no effect on alertness was reported, effects on calmness were replicated in the 2003 study following both the 1000 mg (1 and 6 h post-intervention) and 1600 mg doses (all time points) compared to the placebo [46]. Under stressful conditions induced by the DISS battery, Kennedy and colleagues showed ameliorated negative mood effects of DISS after 600 mg lemon balm, but not at 300 mg, relative to a starch-based placebo [47]. Finally, Kennedy and colleagues assessed several doses of lemon balm (LB) combined with valerian (V), including 600 mg (240 mg LB, 360 mg V), 1200 mg (480 mg LB, 720 mg V), and 1800 mg (720 mg LB, 1080 mg V) [48]. Here, the lowest dose showed no effect on VAS calmness or alertness, but did attenuate the increase in STAI scores induced by the DISS at 3 and 6 h post-DISS. In contrast, the highest dose produced a small but significant increase in state anxiety 1h post-DISS, which continued to trend for the full 6 h follow-up period.

Another stress-based task, known as the Multi-tasking Framework battery (MTF), was used to investigate the anti-stress effects of lemon balm (standardised to a 2% RA content but no phytochemical screening reported) administered in foodstuffs [29]. In iced tea containing 300 mg lemon balm, significantly lower STAI state anxiety and higher working memory scores emerged compared to a placebo fruit-sweetened beverage 1 and 3 h post MTF. However, 600 mg lemon balm iced tea showed higher accuracy on mathematical processing scores but with higher state anxiety scores. Effects of yoghurt emulsions containing lemon balm were less clear, and the authors suggest that food matrices can affect the bioavailability of lemon balm and components, which, in turn, influences mood and cognitive function responses.

With regard to chronic supplementation, one study investigated a 4-day intervention of a combined tablet containing 180 mg of lemon balm (phytochemical screening using HPLC revealed 11.862 RA milli-absorbance units), 270 mg of valerian, 270 mg of butterbur, and 270 mg of passionflower in alleviating stress induced by the Trier Social Stress Test (TSST) in a male-only population [49]. Following the intervention, on day four, the State–Trait Anxiety Inventory (STAI) was filled out before the TSST, immediately after the TSST, and then again 0.5 and 1.5 h following the TSST. The TSST induced a significant increase in self-reported state anxiety, which was significantly attenuated after 30 min following the lemon balm combination tablet compared to the control conditions (unspecified placebo and no treatment). Similar to that seen in female adolescents, young adult women (aged 18 and over) experiencing PMS also had significant improvements in quality-of-life (QOL) scores after supplementing 500 mg of lemon balm capsules twice daily relative to a starch-based placebo, over two consecutive menstrual cycles. These QOL scores showed a reduction in depression, anxiety, and sleep disturbance ratings [50]. Interestingly, when supplementing a combination treatment of 250 mg of lemon balm alongside 250 mg of lavender twice daily over two consecutive menstrual cycles, there were no significant effects on mood or sleep scores relative to the placebo, which the authors suggest may be attributed to the lower daily dose of lemon balm received in the combined treatment (500 mg) compared to the lemon balm-only treatment (1000 mg). Still focusing on young women but this time recruiting participants with post-partum blues, 1500 mg lemon balm over 10 days significantly improved low mood, with the most significant mood improvements seen between days 3 and 5 and with beneficial effects that persisted for 4 days after treatment cessation [51].

The effects of lemon balm on cognition and mood were mixed in trials involving young adults. The variation in duration of dosing, ranging from just a single dose [45] to four days [29]; the use of laboratory stress tests that bear little resemblance to real-life daily stress; and cognitive demand from differing experimental paradigms varying greatly mean that the results should be interpreted carefully.

**Table 2:** Key characteristics of included experimental trials using lemon balm and botanical combinations in young adults.

Citation	Design	Participants			Intervention		Design Method	Findings
		No.	Age	Population	Duration	Dosage (E: Experimental Group, C: Control Group)		
Beihaghi et al. (2019) [51]	Triple-blind RCT (not mentioned how triple-blind)	60	18–35	Healthy post caesarean section	14 days (10-day treatment)	E: 3 × 0.5 g lemon balm capsules/d (n = 30) C: 3 × 0.5 g matching unnamed placebo capsules/d (n = 30)	*Baseline measurements:* No sign of depression at entry (BDI < 10) At d 0, 3–5, 10, and 14: *Mood measurements*: EPDS (post-partum blues) *Tolerability:* Researcher checklist where AEs also assessed	Group E (at d 3–5 compared to Group C): ↓ Post-partum blues in time-dependent response, particularly at day 3–5 and persisting until d 14 Dropouts (not mentioned) No AEs developed after 10 d
Kennedy et al. (2002) [45]	Double-blind cross-over RCT	20	18–22	Healthy	Single dose with 7-day washout between groups	E1: 2 × 0.15 g lemon balm capsules/d E2: 4 × 0.15 g lemon balm capsules/d E3: 6 × 0.15 g lemon balm capsules/d C: 0 mg matching placebo capsules/d In vitro human occipital tissue IC_50_ cholinergic binding activity	*Baseline assessment:* Exclusion of smokers (may influence at the nicotinic receptor level) Timepoints 0, 1, 2.5, 4 and 6 h: *Cognitive assessment:* computerised CDR battery and serial subtraction task (memory and attention) *Mood assessment:* BL-VAS 100 mm (calmness, alertness, contentedness)	Groups E1–E3 (compared to Group C): ↑ Attention accuracy in E2 only at all timepoints post-intervention ↓ Performance on word recognition and spatial memory tasks at various timepoints for all three doses ↑ Calmness in E1 and E3 at 1 h, and in E1 at 2.5 h ↓ Alertness in E3 at all timepoints and E1 at 6 h ↓ Binding of nicotinic and muscarinic receptors Dropouts (n = 0)
Kennedy et al. (2003) [46]	Double-blind cross-over RCT	20	18–23	Healthy	Single dose with 7-day washout between groups	E1: 3 × 0.2 g lemon balm capsules/d E2: 5 × 0.2 g lemon balm capsules/d E3: 8 × 0.2 g lemon balm capsules/d C: 0 g inert placebo capsules/d Phytochemical screening prior to capsule preparation assessing human acetylcholine inhibition and receptor-binding properties	*Baseline assessment: * Chosen IC_50_ concentrations for nicotinic and muscarinic receptor displacement (0.18 and 3.47 mg ml^−1^, respectively). Exclusion of smokers (may influence at the nicotinic receptor level) Timepoints 0, 1, 3, and 6 h: *Cognitive assessment:* Computerised CDR battery and RVIP (memory and attention) *Mood assessment:* BL-VAS 100 mm (calmness, alertness, contentedness)	Groups E1–E3 (compared to Group C): No cholinesterase inhibitory properties detected ↑ Quality of memory performance in E3 at 3 and 6 h, and E1 at 6 h ↓ Speed of processing across memory and attention tasks at various timepoints for all three doses ↑ Calmness in E2 at 1 h and 6 h, and in E3 at 1, 3, and 6 h Dropouts (n = 0)
Kennedy et al. (2004) [47]	Double-blind cross-over RCT	18	*Age* *range* *not* *provided* *in paper* *M* = 29	Healthy	Single dose with 7-day washout between groups	E1: 2 × 0.15 g lemon balm capsules/d E2: 4 × 0.15 g lemon balm capsules/d C: 0 g inert placebo capsules/d	Timepoints 0 and 1 h: *Cognitive stress task:* DISS (20 min duration) *Mood assessment:* BL-VAS 100 mm (alertness, contentedness, calmness)	Groups E1–E2 (at 1 h compared to Group C): Attenuation of negative mood induced by DISS after E2, particularly increased calmness and reduced alertness ↑ Speed of mathematical processing after E1, with no compromise on accuracy Dropouts (n = 0)
Kennedy et al. (2006) [48]	Double-blind cross-over RCT	24	*Age* *range* *not* *provided* *in paper* *M = 23.48*	Healthy	Single dose with 7-day washout between groups	E1: 3 × 0.08 g lemon balm and 0.12 g valerian capsules/d E2: 6 × 0.08 g lemon balm and 0.12 g valerian capsules/d E3: 9 × 0.08 g lemon balm and 0.12 g valerian capsules/d C: 0 g inactive placebo capsules/d	*Baseline assessment* Exclusion of smokers (may influence at the nicotinic receptor level) Timepoints 0, 1, 3, and 6 h per block: *Cognitive stress task:* DISS (20 min duration). *Mood assessments: * BL-VAS 100 mm (alertness, contentedness, calmness) and STAI (state and trait anxiety)	Group E1–E3 (compared to Group C): Attenuation of state anxiety induced by DISS after E1 at 3 and 6 h ↑ State anxiety after E3 at 1 h and trend of persisting up to 6 h ↓ Trait anxiety at 1 h in E1 with a trend up to 3 h ↓ Stroop performance at 3 h and 6 h in E1, at all time points for E2, and at 1 h for E3 Dropouts (n = 0)
Meier et al. (2018) [49]	Double-blind RCT	70	18–45 *M* = 26.07	Healthy males	4 days	3 tablets taken p/d for 3 d and 2 tablets taken on d 4: E1: 1 tablet contained “Ze 185” 0.06 g lemon balm, 0.09 g valerian, 0.09 g Butterbur and 0.09 g passionflower tablet/d (n = 23) C1: unnamed placebo tablet (n = 23) C2: no treatment (n = 24) Phytochemical screening of lemon balm: HPLC revealed 11.862 RA mAU	*Baseline assessment:* *Tolerability:* HR, BP, serum markers, and AEs *Anxiety measurement:* STAI *Physiological stress measurement:* cortisol at d 4: *Cognitive stress task:* TSST computerised battery *Physiological stress measurement:* 11 × saliva (cortisol measurements at −50, −35, −20, −10 min pre-TSST and 0, 10, 20, 30, 45, 60, 90 min post TSST) *Stress assessment:* 5 × STAI (pre-TSST, −45 to TSST completion, immediately post TSST and 30, 90 min post TSST) *Tolerability:* AEs recorded, HR, BP, serum markers	Group E1 (following TSST on d 4 compared to Group C1 and C2): Homogenous in cortisol levels where TSST ↑ cortisol at d 4, but 10 cortisol non-responders ↓ State anxiety immediately after TSST and up to 30 min after No serious AEs developed, headaches and flatulence but recovered by study cessation (n = 3) Dropouts (n = 2 after randomisation but before receiving study medication)
Scholey et al. (2014) [29]	2 × Double-blind crossover RCT	RCT 1: 25 RCT 2: 21	RCT 1: 18–39 *M* = 25.3 RCT 2: 21–30 *M =* 23.6	Healthy	Single dose with 7-day washout between groups	RCT 1: E1: 1 × 0.3 g lemon balm (RA > 6%) and natural fruit sweetener beverage/d of 480 mL E2: 1 × 0.6 g lemon balm (RA > 6%) and natural fruit sweetener beverage/d of 480 mL E3: 1 × 0.6 g lemon balm (RA > 6%) and artificial sweetener blend of 480 mL C: 1 × 480 mL artificial sweetener placebo beverage/d RCT 2: E1: 1 × 250 g yoghurt with 0.3 g lemon balm (RA > 6%) and natural fruit sweetener E2: 1 × 250 g yoghurt with 0.6 g lemon balm(RA > 6%) and natural fruit sweetener E3: 1 × 250 g yoghurt with 0.6 g lemon balm (RA > 6%) and artificial fruit sweetener C: 1 × 250 g yoghurt with artificial sweetener placebo/d	*Baseline assessment:* Pilot study of 5 stressed young adults (independent of RCT 1 and 2) found >2% RA bioavailability in 1.8 g lemon balm and 200 mL fruit sweetener drink peaked at 1 h in serum biomarkers when trialling cognitive and mood batteries RCT 1: Timepoints 0, 1, and 3 h: *Cognitive stress task:* MTF computerised battery (psychomotor tracking, memory, attention) *Mood assessments: * STAI (state and trait anxiety), DASS (stress) *Physiological Stress marker:* 1 × saliva sample (cortisol) at 12 p.m. or 4 p.m. RCT 2: Timepoints 0, 1, and 3 h *Cognitive stress task:* MTF adapted from DISS and 20 min duration (psychomotor, memory, attention) *Mood assessments:* BL-VAS 100 mm (stress and fatigue)	RCT 1: Group E1–E3 (compared to Group C): Attenuation of MTF induced anxiety after E1 at 1 h and 3 h ↑ State anxiety at 1 h in E3 ↑ Cognitive ability in maths processing at 1 h in E2, tracking in E2, and working memory scores in E3 at both timepoints Elevated cortisol in C at 1 h not evident in E groups Dropouts (n = 0) RCT 2: Group E1–E3 (compared to Group C): ↑Alertness after E1 at both timepoints ↑ Fatigue at 1 h in E2 and E3, but fatigue scores dropped at 3 h for E2 ↑ Maths performance in E1 at 3 h, and immediate word recall at 1 h for E1 and E2 No AEs reported in either RCT

BDI, Beck’s Depression Inventory; BL-VAS, Bond–Lader Visual Analogue Mood Scale; BP, blood pressure; CDR, cognitive drug research; DISS, defined intensity stressor stimulation; EDPS, Edinburgh Postnatal Depression Scale; HPLC, high-performance liquid chromatography; HR, heart rate; mAU, milli-absorbance units; MTF, multi-tasking framework; RVIP, rapid information processing task; RA, rosmarinic acid; STAI, State and Trait Anxiety Inventory; TSST, Tier Social Stress Test; ↑, increased; ↓, decreased.

#### 2.3.3. Middle-Aged Adults

Lemon balm studies with middle-aged adults aged up to 55 years are summarised in Table 3. Two weeks of 1000 mg daily supplementation of lemon balm capsules showed a significant decrease in the frequency of heart palpitations, although not the severity of symptoms, in adults with benign heart palpitations relative to a breadcrumb-based placebo [52]. In addition, anxiety, sleep problems, and depression were significantly reduced after the lemon balm supplementation. Another study in 16 depressed patients taking 2000 mg lemon balm (phytochemical screening of phenolic and flavonoid content, 4.88 GA/g and 4.28 RU/g, respectively) daily for 8 weeks showed a significant anti-depressant effect relative to baseline scores, similar to the anti-depressant response produced by 10 mg of fluoxetine supplementation [53].

Several studies have been interested in exploring the sleep-enhancing effects of lemon balm alone and in combination with other herbal supplements. Women with menopausal symptoms showed significant improvements in sleep quality after 4 weeks of 160 mg daily lemon balm, coupled with 320 mg valerian, in comparison to a starch-based placebo [54]. Similarly, 8 weeks of 500 mg lemon balm with a fennel extract showed positive QOL changes in menopausal women with sleep problems, with the greatest effect on their vasomotor symptoms relative to both a starch-based placebo and a positive control of 30 mg citalopram [55].

In a study of healthy adults aged 20 to 70 with mild sleep problems, 240 mg lemon balm taken together with 360 mg valerian for 30 days failed to produce any significant changes in self-reported VAS sleep quality compared to placebo treatment [56]. However, 23 out of 98 participants verbally reported positive improvements in sleep quality during the study follow-up. Other studies have investigated healthy adults with moderate sleep problems (as defined by the Pittsburgh Sleep Quality Index (PSQI), score of 6–15) and found that 6 weeks of a daily formulation containing 80 mg of lemon balm (standardised to ≥2% RA content but no phytochemical screening reported) induced significant improvements in sleep quality and better daytime functioning using the modified Athens Insomnia Scale (mAIS) relative to a placebo [57]. Daytime benefits included the perception of feeling refreshed upon awakening and overall well-being. Furthermore, activity trackers and sleep diaries were used to estimate sleep quality based on sleep–wake patterns but no differences relative to placebo were observed. Similarly, a daily dose of 400 mg lemon balm (standardised to 17–23% hydroxycinnamic acid and analysed for RA content but no phytochemical screening reported) taken for 3 weeks in 100 middle-aged adults with moderate emotional problems (depression ≥ 14, anxiety ≥ 10 or stress ≥ 19, DASS-42) or sleep-quality problems (PSQI > 5) showed enhanced sleep-quality improvements amongst a host of other improvements in various mood measures, including DASS-42, PANAS, and the Warwick–Edinburgh Mental Well-Being Scale relative to an unnamed matched placebo [58].

In those with mild-to-moderate insomnia (Insomnia Severity Index (ISI), entry 8–21) 40 adults taking a formulation containing 240 mg of lemon balm experienced enhanced sleep quality, longer duration of sleep, reduced latency to sleep onset, and fewer night-time wakings, according to their 2-week sleep diary reports, compared to placebo-treated participants [59]. Similarly, 23 adults with diagnosed insomnia (ISI entry > 7) responded positively to a 4-week treatment with 1000 mg lemon balm combined with 400 mg lavender; significant reductions in BAI anxiety, BDI depression, and ISI insomnia scores relative to a starch-based placebo were seen [60].

Overall, the effects of lemon balm on mood and sleep quality are still unclear in middle-aged adults where several studies used a combination of herbal treatment and/or supplements. Thus, the synergistic effects between lemon balm and other herbal supplements may be responsible for the beneficial outcomes, warranting caution in interpreting lemon balm’s independent efficacy.

**Table 3:** Key characteristics of included experimental trials using lemon balm and botanical combinations in middle-aged adults.

Citation	Design	Participants			Intervention		Design Method	Findings
		No.	Age	Population	Duration	Dosage (E: Experimental Group, C: Control Group)		
Alijaniha et al. (2015) [52]	Double-blind RCT	55	18–60 *M* = 42	Healthy with benign heart palpitations	2 weeks	E: 2 × 0.5 g lemon balm capsules/d (n = 28) C: 2 × 0.5 g breadcrumb capsules/d (n = 27)	*Baseline measurements:* Safety serum markers, ECG, and self-report heart palpitation perception At w 0 and 2: - Heart palpitations frequency and intensity VAS-10 cm - GHQ-28 (well-being, anxiety, sleep)	Group E (at w 2 compared to Group C): ↓ GHQ-28 (only anxiety and insomnia) ↓ Heart palpitation frequency only No AEs Dropouts (n = 8)
Araj-Khoadei et al. (2020) [53]	Double-blind RCT, no placebo	45	18–65 *M* = 37	Diagnosed depression (DSM-V criteria)	8 weeks	E1: 4 × 0.5 g lemon balm capsules/d (n = 16) E2: 4 × 0.5 g lavender capsules/d (n = 17) C: 2 × 10 mg/d fluoxetine (n = 17) Phenolic (Folin–Ciocalteu’s reagent/gallic acid) and flavonoid (rutin) characterisation of herbals revealed lemon balm constituents phenolic at 4.88 mg GA/g and flavonoid at 4.28 RU/g	*Baseline measurements:* HAM-D for mild to moderate depression (score range 8–24) At w 0, 2, 4, and 8: - HAM-D (depression) - AE self-reports	Group E1 (at w 8 compared to Group E2 and Group C): Homogenous across all groups in ↓ depression ratings No AEs Dropouts (n = 2)
Bano et al. (2023) [58]	Double-blind RCT	100	18–65 (*M = 31)*	Healthy with moderate mood or sleep problems	3 weeks	E: 2 × 0.2 g lemon balm capsules/d with phospholipid (sunflower) carrier (n = 52) C: 2 × 0.2 g matched unnamed placebo capsules/d (n = 48)	*Baseline measurements:* Moderate depression, anxiety, or stress (DASS-42; ≥14, ≥10, and ≥19, respectively) At w 0 and 3: *Sleep and mood assessments:* PSQI (sleep quality), DASS-42 (depression, anxiety, stress), PANAS (mood), WEMWBS (well-being), QoL (quality of life) *Tolerability:* AEs reported throughout study	Group E (at w 3 compared to Group C): ↓ PSQI ↓ DASS-42 in depression, anxiety, and stress ↑ PANAS positive affect ↓ PANAS negative affect ↑ WEMWBS ↑ QoL No AEs Dropouts (n = 0)
Bongartz et al. (2019) [57]	Double-blind RCT	50	23–64 *M* = 46	Healthy, with moderate sleep problems	6 weeks	E: “IQP-AO-101” sachet/d in 100 mL water containing 0.08 g lemon balm, 0.3 g asparagus extract, 0.01 g zinc, 0.03 g saffron extract, 0.06 g vitamin C, 0.03 IU vitamin E (n = 25) C: matched placebo containing excipient sachet/d in 100 mL water (n = 25)	*Baseline measurements:* Sleep study entry (PSQI 6–15) and safety serum markers, HR, BP At w 0, 1, 4, and 6: *Sleep and mood assessments:* mAIS (sleep), POMS-65 (mood), FAIR-2 (attention), sleep diary (sleep) AE self-reports At w 0 and 4: *Sleep assessment:* Fitbit Flex 2 activity tracker (HR variability)	Group E (at w 6 compared to Group C): ↓ mAIS, nighttime, and daytime parameters. No AEs Dropouts (n = 0)
Cerny and Schmid (1999) [56]	Double-blind multi-centred parallel RCT	98	20–70 *M* = 33	Healthy, with mild sleep problems	30 days	E: 3 × 0.08 g lemon balm and 0.12 g valerian tablets/d (n = 66) C: matched placebo without herbals 0.6 g tablets/d (n = 32)	*Baseline measurements*: verbal report of sleep problems entry and safety serum markers, BP, HR d 0 and 30: *Sleep and wellbeing* *assessments:* VAS 100 mm and verbal report *Tolerability and AEs: * self-report (5-point rating) and physical examination	Group E (at d 30 compared to Group C): Positive trend in VAS sleep quality homogenous across groups ↑ Sleep quality (verbal report) ↑ Tolerability self-report acceptable to excellent evaluation No AEs Dropouts (n = 2)
Lemoine et al. (2019) [59]	Open-label pilot study	40	20–75 *M* = 33	Healthy, with mild-to-moderate sleep complaints	2 weeks (with 1-week follow-up)	E: 2 × 0.12 g lemon balm, 0.001 g melatonin, 0.00042 g vitamin B6, 0.0084 g Californian poppy extract, 0.075 g passionflower extract capsules/d (n = 40)	*Baseline measurements*: ISI (sleep entry 8–21), 1-week run-in fixed sleep schedule, restricted caffeine, and completed sleep diary w 2, 3, and follow-up w 4: *Sleep assessment:* Sleep diary recording sleep quality (0–10, 10 = good/very good sleep), time to fall asleep, nighttime wakings, daytime naps, and wake-up time *Tolerability:* AEs reported throughout study	Group E (at post-treatment and follow-up compared to baseline): ↑ Sleep quality and sleep duration ↓ Sleep onset latency ↓ Nighttime wakings and daytime fatigue No reported AEs Dropouts (n = 0)
Ranjbar et al. (2018) [60]	Double-blind RCT	45	18–60 *M* = 39	Insomnia (ICSD II criteria), with co-morbid depression and anxiety	4 weeks	E: 3 × 0.5 g capsules/d to amount 1 g lemon balm and 0.4 g lavender (n = 23) C: 3 × 0.5 g starch-based capsule/d (n = 22)	*Baseline measurements:* Homogenous for sleep problems (ISI > 7), depression (BDI > 10I), and anxiety (BAI > 7). W 0 and 4: *Sleep and mood assessment:* ISI (sleep) and BAI (anxiety) *Tolerability:* AE self-reports w 0, 2, and 4: *Mood assessment:* BDI (depression).	Group E (at w 4 compared to Group C): ↓ Insomnia ↓ Anxiety ↓ Depression Dropouts (n = 2)
Shirazi et al. (2021) [55]	Double-blind RCT	60	43–60 *M* = 52	Post-menopausal women (hormone and period absence confirmation) with sleep disorders	8 weeks	E: 1 × 0.5 g lemon balm and fennel fruit extract capsule/d (n = 20) C1: 1 × 0.03 g citalopram capsule/d (n = 20) C2: 0.5 g starch-based placebo (n = 20)	*Baseline measurements:* Sleep disturbance entry (PSQI ≥ 5) w 0 and 8: *Wellbeing and sleep assessment: * MENQOL (menopause quality of life) *Tolerability:* Self-reported AEs at w 8	Group E (at w 8 compared to Groups C1 and C2): ↑ Quality of life in improved vasomotor symptoms ↑ Quality of life in improved psychomotor–social, physical, and sexual domains No AEs, whereas C1 had reported headaches Dropouts (n = 0)
Taavoni et al. (2013) [54]	Triple-blind RCT	100	50–60 *M* = 54	Post-menopausal women with sleep disorders	4 weeks	E: 2 × 0.08 g lemon balm and 0.16 g valerian capsules/d (n = 50) C: 2 × 0.05 g starch-based capsules/d (n = 50)	*Baseline measurements:* Sleep disturbance entry (PSQI ≥ 5) w 0 and 4: *Sleep assessment:* PSQI (sleep quality) *Tolerability:* verbal report of AEs at w 4	Group E (at w 4 compared to Group C): ↑ Sleep quality in both groups, but sig. greater in group E No AEs

AE, adverse events; BAI, Beck’s Anxiety Inventory; BDI, Beck’s Depression Inventory; BP, blood pressure; ECG, electrocardiogram; FAIR-2, Frankfurt Attention Inventory; GHQ-28, General Health Questionnaire; HAM-D, Hamilton Depression Scale; HR, heart rate; ISI, Insomnia Severity Index; mAIS, modified Athens Insomnia Scale; PANAS, positive and negative affect; POMS-65, Profile of Mood States; PSQI, Pittsburgh Sleep Quality Index; QoL, quality of life; VAS, Visual Analogue Scale; WEMWBS, Warwick–Edinburgh Mental Well-Being Scale, ↑, increased; ↓, decreased.

#### 2.3.4. Older Adults

The effects of lemon balm supplementation in older adults aged over 55 are summarised in Table 4. Several RCTs have investigated the calmative effects of lemon balm on adults experiencing psychological symptoms produced by their ongoing heart condition. For instance, in 35 older adults with chronic but stable angina, 8-week supplementation of 3000 mg lemon balm daily was associated with lower ratings of anxiety and stress (assessed using the DASS-21) as well as reduced sleep disturbance (using PSQI) relative to a cornstarch placebo [61]. Similar findings were reported at a much lower dose of 1500 mg (phytochemical screening of phenolic and flavonoid, 4.88 GA/g and 4.28 RU/g, respectively) taken for 7 days, where sleep quality was improved (assessed with SMHSQ) and anxiety reduced (using the HADS) in 40 post-cardiac surgery patients relative to a wheat starch placebo [62]. Further evidence for a beneficial effect on anxiety in those with heart problems was reported in a study of older adults with cardiac problems (n = 36), who scored lower on transient anxiety (DASS-21) after 5 min of lemon balm (phytochemical screening using quality assay revealed 24.4% beta caryophyllene, 8.6% geranial, 6.9% 1,8-cineole, 6.7% neral, 5.8% dehydroaromedendrene, 4.8% thymol, and 3.8% α-Pinene components) inhalation (2 drops) on two occasions 90 min apart relative to a sunflower oil control [63]. In addition, the authors suggested that hemodynamic changes seen in mean arterial pressure scores support the notion of anti-stress actions following lemon balm treatment.

Three studies explored the effects of lemon balm treatment on older adults with and without dementia experiencing agitation and/or cognitive decline. Firstly, when 20 older adults inhaled 60 drops of lemon balm daily, significant improvements in cognitive performance relative to baseline were seen in both the ADAS-Cog and the Clinical Dementia Rating scores [64]. In a similar study [65] using the Neuropsychiatric Inventory (NPI) and the Cohen–Mansfield Agitation Inventory (CMAI) to assess agitated and irritable behaviour in nursing home residents with and without dementia, inhalation of two drops of lemon balm oil (phytochemical screening revealed beta caryophyllene, germacrene D, citral, and geraniol components) daily for two weeks had no effect on agitation levels relative to a sunflower oil placebo in residents with dementia. However, residents without dementia showed significant reductions in physical non-aggressive behaviour and irritability frequency and severity following lemon balm treatment relative to the sunflower oil placebo). The final double-blind RCT reviewed here used 24 weeks of 500 mg daily lemon balm in older adults with mild dementia due to Alzheimer’s disease. Participants showed significant improvement in irritability scores, assessed using the NPI, compared to an unnamed placebo. However, no differences in cognitive performance measured by the ADAS-Cog were seen between baseline and the 24-week follow-up [66].

From the available studies, lemon balm appears to produce significant cognitive and mood-enhancing benefits in older adults (with and without other health conditions) after 7 days of supplementation. However, results are less clear for mood and cognitive changes in older adults with neurodegenerative conditions, although further long-term (>16 weeks) studies are required here before actions in this population can be determined.

**Table 4:** Key characteristics of included experimental trials using lemon balm and botanical combinations in older adults.

Citation	Design	Participants			Intervention		Design Method	Findings
		No.	Age	Population	Duration	Dosage (E: Experimental Group, C: Control Group)		
Akhondzadeh et al. (2003) [64]	Double-blind RCT	42	65–80 *M* = 73	Mild-to-moderate AD (NINCDS/ADRDA diagnosis)	16 weeks	E: 60 lemon balm drops/d (dose unspecified) (n = 20) C: 60 drops/d matching placebo (unspecified) (n = 15)	*Baseline measurements:* Cognitive decline assessment ADAS-cog ≥ 12 and CDR-SB ≤ 2 *Tolerability:* ECG, HR, and BP w 0 to 16 (every 2 weeks): *Cognitive and mood assessments:* ADAS-cog and CDR-SB *Tolerability:* ECG, HR, and BP and self-report /observed AEs	Group E (at w 16 compared to Group C): ↑ Cognition (ADAS-cog and CDR-SB) No serious AEs reported Dropouts (n = 7)
Haybar et al. (2018) [61]	Double-blind RCT	73	40–75 *M* = 59	Adults experiencing CSA	8 weeks	E: 3 × 1 g lemon balm capsules/d (n = 35) C: 3 × 1 g cornstarch placebo capsules/d (n = 38)	*Baseline measurements*: 3-day habitual diet and physical activity level (IPAQ) w 0 to 8: *Sleep and mood assessments: * DASS-21 (anxiety, depression, and stress), PSQI (sleep quality)	Group E (at w 8 compared to Group C): ↑ Sleep quality, sleep duration, and sleep efficiency ↓ Depression, anxiety, and stress Dropouts (n = 8)
Noguchi-Shinohara et al. (2020) [66]	Double-blind RCT (24 weeks) followed by 24-week treatment, no placebo	20	>60 *M* = 89.3	Mild dementia due to AD (n = 20) (MMSE screened between 20 and 26)	48 weeks	*Part 1:* 24-week double-blind 500 mg lemon balm capsules or unnamed placebo capsules/d *Part 2:* 24-week 500 mg lemon balm only/d	w 0, 8, 16, 24, 32, 40, and 48: *Tolerability:* ECG, urinalysis, haematology, blood chemistry, chest X-ray, HR, and BP w 0, 16, and 24: *Mood and cognitive assessments: * ADAS-Cog (memory), NPI (agitation), MMSE (executive function, memory), DAD (executive function)	↓ Agitation and lability in lemon balm group No difference in cognitive performance between interventions
Soltanpour et al. (2019) [62]	Double-blind RCT	80	30–70 *M* = 58	Post-surgery cardiac patients experiencing anxiety and sleep disturbance	7 days	E: 3 × 0.5 g lemon balm capsules/d (n = 40) C: 3 × 0.5 g wheat starch placebo capsules/d (n = 40) *Herbal assay:* phenolic (Folins–Ciocalteu’s agent and gallic acid) and flavonoid (rutin) components revealed phenolic and flavonoid, 4.88 GA/g and 4.28 RU/g, respectively	*Baseline measurements:* No considerable sleep disorder prior to surgery (PSQI < 28) d 0 to 7: *Sleep and mood* *assessment:* SMHSQ (sleep quality), HADS (anxiety)	Group E (at d 7 compared to Group C): ↑ Sleep quality ↓ Anxiety No AEs Dropouts (n = 0)
Veiskaramian et al. (2021) [63]	Double-blind RCT	70	35–65 *M =* 57	ACS patients experiencing stress	1 day	E: 2 phases (with 90 min interval). Each phase = 2 drops lemon balm oil for 10 min (n = 36) C: 2 phases (with 90 min interval). Each phase = 2 drops sunflower oil for 10 min (n = 34) Phytochemical screening of lemon balm: 24.4% beta caryophyllene, 8.6% geranial, 6.9% 1,8-cineole, 6.7% neral, 5.8% dehydroaromedendrene, 4.8% thymol, and 3.8% α-Pinene components),	*Baseline measurements*: Cardiac markers: MAP > 70 mm Hg and HR > 60 Stress (DASS-21 > 19) and chest pain (10 cm VAS ≥ 3cm) Timepoints, t1 to 6: *Stress assessment:* DASS-21 (stress), MAP taken before (t1), 5 mins (t2), and 15 mins (t2) after phase 1. Interval of 90 min followed by t4–6 for phase 2. Threat perception scale as secondary stress measure.	Group E (compared to Group C): ↓ Acute stress in t2 and t5 ↓ HR t2 and t5 ↓ MAP t2 ↓ Chest pain across timepoints Within group E ↓ in threat perception at t6 vs. t1 No AEs Dropouts (n = 0)
Watson et al. (2019) [65]	Counter-balanced repeated measures, double-blinded design	49	>65 *M* = 89.3	Dementia (n = 39) and non-dementia (n = 10) living in residential care homes	2 weeks (with 2-week washout periods))	E1: 2 drops lemon balm oil/d E2: 2 drops lavender oil/d C: 2 drops placebo sunflower oil oil/d *All treatments were* *applied to a cloth and attached to participant clothing for 2 h* *inhalation blocks* Phytochemical screening of lemon balm revealed beta caryophyllene, germacrene D, citral, and geraniol components	*Baseline measurements: * Mood and cognitive level entry: MMSE (>10 cognition) and NPI (≥6 agitation frequency) w 0–2, 4–6, and 8–10: *Mood & cognitive assessments*: CMAI (physical non-aggressive behaviour) and NPI (agitation)	Group E1 (compared to E2 and Group C): ↓ Agitation and physical non-aggressive behaviour in non-dementia groups ↑ Agitation compared to E2 in dementia group Dropouts (n = 6)

ACS, acute coronary syndrome; ADAS-Cog, Alzheimer’s Disease Assessment Scale—Cognitive subscale; AD, Alzheimer’s disease; ADRDA, Alzheimer’s Disease and Related Disorders Association; CDR-SB, Clinical Dementia Rating—Sum of Boxes; CSA, chronic stable angina; CMAI, Cohen–Mansfield Agitation Inventory; DAD, Disability Assessment for Dementia; DASS-21, Depression, Anxiety & Stress Scale; HADS, Hospital Anxiety & Depression Scale; HR, heart rate; IPAQ, International Physical Activity Questionnaire; MAP, mean arterial pressure; NINCDS, National Institute of Neurological & Communicative Disorders; NPI, Neuropsychiatric Inventory; MMSE, Mini-Mental State Examination; PSQI, Pittsburgh Sleep Quality Index; SMHSQ, St. Mary’s Hospital Sleep Questionnaire, ↑, increased; ↓, decreased.

#### 2.3.5. Across the Lifespan

Table 5 summarises the study design and findings from studies where lemon balm treatment has been administered in individuals across an age range of 18–75 years. A 15-day open-label trial showed 600 mg lemon balm (phytochemical screening revealed 7.95 ± 0.29% RA; 18.5 ± 0.63% total hydroxycinnamates; flavonoids, including 0.23 ± 0.01% hesperidin, 0.46 ± 0.04% luteolin-3-glucuronide, and 0.69 ± 0.04% total flavonoids; and triterpenes, including 0.22 ± 0.03 oleanolic acid and 0.64 ± 0.09% ursolic acid) capsules taken daily improved anxiety manifestations (AMs) and anxiety-associated symptoms (AASs) using the Free-Rating Anxiety Scale in 20 healthy adults with mild anxiety and insomnia problems [67]. AMs such as agitation were noticeably reduced post lemon balm, as well as AAS ratings of guilt and inferiority feelings relative to baseline scores. In addition, significant reductions in insomnia and depression ratings (Hamilton Depression Scale) were seen relative to baseline. Furthermore, using the Clinical Global Rating Scale score of ≤2 to indicate a lack of relapse post treatment, recovery in 14/20 respondents with anxiety and 17/20 respondents with insomnia symptoms was seen.

Chehroudi and colleagues [68] randomised 36 burn patients to receive lemon balm tea at a daily dose of 5000 mg or a black tea control for 20 days. Those in the lemon balm group evidenced a significant reduction in depressive symptoms compared with the 18 patients in the control group. In addition, Kettle Anxiety Questionnaire scores were significantly reduced following lemon balm relative to the control. In the lemon balm group, 12 (out of 18) adults reported a PSQI score indicating sleep problems (PSQI > 5), 50% of which moved into the bracket of no reported sleep problems compared to only 1 person in the placebo group (10/18 reporting PSQI > 5 at baseline). Two further 3-day-long studies investigating 92 and then 94 adults with cardiac problems found that 3 drops of lemon balm inhaled for 30 min twice daily was associated with significant improvements in anxiety or depression ratings. In the first trial, STAI anxiety scores were significantly decreased in the lemon balm group post intervention relative to a sesame oil control [69]. In their second trial, sleep disorder, sleep efficacy, and sleep VAS scores such as daily napping significantly improved in the group receiving lemon balm compared to the control group [70].

In summary, lemon balm shows reasonable evidence of reducing mild psychological disturbance, alleviating sleep problems, and improving mood and cognitive ability across the lifespan. Whilst the studies reviewed in this section were mostly robust in design, some lacked appropriate control/placebo conditions, which makes interpretation difficult. In addition, due to the inconsistency in the measures chosen, in terms of both dose and length of study, it is challenging to determine the efficacy of lemon balm when assessing its psychological effects across the lifespan.

**Table 5:** Key characteristics of included experimental trials using lemon balm and botanical combinations across the lifespan.

Citation	Design	Participants			Intervention		Design Method	Findings
		No.	Age	Population	Duration	Intervention (E: Experimental Group, C: Control Group)		
Cases et al. (2011) [67]	Open-label pilot	20	18–70	Healthy with mild anxiety and sleep problems (DSM-IV-TR)	15 days	E: 2 × 0.3 g lemon balm capsules/d (n = 20) Phytochemical screening of lemon balm: 7.95 ± 0.29% RA; 18.5 ± 0.63% total hydroxycinnamates; flavonoids, including 0.23 ± 0.01% hesperidin, 0.46 ± 0.04% luteolin-3-glucuronide, and 0.69 ± 0.04% total flavonoids; triterpenes, including 0.22 ± 0.03 oleanolic acid and 0.64 ± 0.09% ursolic acid	d 0 and 15: *Sleep & stress assessments:* FRSA (anxiety manifestations and symptoms), HDRS (insomnia) *Clinical improvement* *for anxiety and sleep assessment:* CGI-I ≤ 2 *Tolerability:* Verbal reports of AEs	Group E (at d 20 compared to d 0): ↓ Agitation as one anxiety manifestation ↓ Eating problems, ↓ feelings of guilt, and ↓ feelings of inferiority as some anxiety-associated symptoms ↓ Initial insomnia and middle insomnia ↑ Clinical improvement in anxiety recurrence and/or sleep problems No reported AEs Dropouts (n = 0)
Chehroudi et al. (2017) [68]	Double-blind RCT	36	n.d.	Hospitalised 2nd- and 3rd-degree burns	20 days	E: 2 × 2.5 g lemon balm tea/d (n = 18) C: 2 × 2.5 g black tea/d (n = 18)	d 0 and 20: *Sleep & mood assessments:* BDI (depression), Kettle’s (anxiety) and PSQI (sleep quality) Serum antioxidants: 5 mL	Group E (at d 20 compared to Group C): ↓ Anxiety ↓ Depression ↑ Sleep quality Homogenous serum antioxidant levels Dropouts (not mentioned)
Lotfi et al. (2019) [69]	Single-blind RCT (only participant blinded)	94	20–75	ACS with co-morbid anxiety symptoms	3 days	E: 2 × 3 drops lemon balm oil/d (n = 45) C: 2 × 3 drops odourless sesame placebo oil/d (n = 47) *All treatments were applied to a cloth and attached to participant clothing for 30 min twice a day for 3 days*	*Baseline* *measurements: * olfactory test with coffee sniffing d 0, 2, and 3: Anxiety measurements: STAI (state and trait anxiety)	Group E (at d 3 compared to Group C): ↓ Anxiety Drop-outs (n = 2)
Lotfi et al. (2020) [70]	Single-blind RCT (only participant blinded)	92	20–75	ACS with co-morbid sleep problems	3 days	E: 2 × 3 drops lemon balm oil/d (n = 45) C: 2 × 3 drops odourless sesame placebo oil/d (n = 47) *All treatments were applied to a cloth and attached to participant clothing for 30 min twice a day for 3 days*	*Baseline* *measurements: * Olfactory test with coffee sniffing D1 and 3: *Sleep assessment:* VSH (sleep quality) in 3 subscales, sleep disorder (0–700 points), sleep efficacy (0–500 points), and daytime napping (0–400 points)	Group E (at d 3 compared to Group C): ↓ Sleep disorders ↑ Sleep efficacy ↓ Daytime napping Dropouts (n = 2)

ACS, acute coronary syndrome; BDI, Beck’s Depression Inventory; FRSA, Free Rating Scale for Anxiety; HDRS, Hamilton Depression Rating Scale; Kettles, Kettles Anxiety Scale; STAI, Spielberger State–Trait Anxiety Inventory; PSQI, Pittsburgh Sleep Quality Index; VSH, Verran–Snyder-Halpern Scale; ↑, increased; ↓, decreased.

## 3. Discussion

Here, we have reviewed the evidence for the actions of lemon balm on psychological well-being across the lifespan. The findings indicate that lemon balm may prevent and help manage symptoms associated with sleep disturbances [41,52,57,58,59,60,61,67,68,69,70], anxiety [61,62,64,66,67,69], low mood [29,45,46,47,48,51,58], and overall quality of life [43,50,55,56,57,58,60]. Mechanistically, several neurochemical pathways, including GABAergic [1,15,20,22,23] and cholinergic pathways [25,26,28], as well as actions on various signalling molecules involved in HPA-axis functioning, enzymes involved in inflammation such as inhibition of cyclooxygenase (COX) [35], and those that modulate NF-κB signalling molecules, support the calmative action of lemon balm [36]. Although pharmacokinetic evidence is limited, it is known that certain metabolites, such as RA and its derivatives, are bioavailable [29,66] and may cross the blood–brain barrier to elicit their effects [1,20]. Across the human trials, lemon balm was consumed in a variety of daily doses of between 80 mg/day and 5000 mg/day, taken either as a single acute dose or with repeated dosing for up to 8 weeks, and in formats including capsules, tablets, and tea, all of which were well tolerated.

Unfortunately, there are no studies to date performing a direct comparison between populations for cognitive, sleep, or mood outcomes, making it challenging to ascertain whether lemon balm may be more efficacious for particular age groups. However, on reviewing the available evidence, there do appear to be consistent benefits to low mood and anxiety across the lifespan, with improvements in mood symptoms reported in children [39,40], young [29,45,46,49,51], middle-aged [52,58,60], and older [61,62,65,66] adults. It should, however, be highlighted that mood outcomes in infants are difficult by nature to measure, particularly in the insistence of colicky infants where neurogenic, gastrointestinal, microbial, and psychosocial factors contribute to symptoms [71]. As such, reduced crying time in colicky infants is arguably an unreliable measure of anxiety, as it is difficult to infer how lemon balm reduces discomfort.

Several studies report consistent findings of improved sleep in children [41], middle-aged adults [52,54,56,58,59,60], and older adults [61,62], although a number of the studies in middle-aged adults combined lemon balm with other herbals, such as valerian, and the use of non-standard self-report measures and the occurrence of placebo effects makes these data more challenging to interpret. The limited evidence in older adults experiencing conditions such as post-surgical anxiety and CSA is compelling, but it should be highlighted that both studies recruited individuals from mid to older adulthood, with a mean age in both studies of below 60. Given that this appears to be a promising avenue of research, more studies focused on sleep outcomes in older adults are warranted. Furthermore, lemon balm may be beneficial for reducing sleep disturbance in older adults with dementia, given that studies showed reduced agitation in dementia populations [65,66]. Moreover, lemon balm may be a therapeutic option for those with sundowning syndrome, which is a condition that affects some individuals with dementia, disrupting circadian rhythms and presenting anxiety and insomnia symptoms [72]. Given the potential calming effects of lemon balm via GABA availability (GABA_A_ receptor affinity, inhibition of GABA-T) [21,24] and inhibition of the acetylcholinesterase enzyme (AChE) [25,26]—a key target site for current management of Alzheimer’s disease [27]—lemon balm may subsequently regulate mood and sleep cycles in this population. As such, there is a clear gap in the existing research for studies exploring lemon balm supplementation for sleep in older adults with cognitive impairment. While the existing evidence for lemon balm and sleep appears promising, it should also be noted that the available sleep trials included subjective measures of sleep, which may instead indicate a placebo effect, particularly in some sleep facets, including sleep latency [73]. Thus, the subjective sleep outcomes should be interpreted with caution given that, in some trials, objective measures of sleep showed no changes following lemon balm supplementation [57]. However, promising evidence for lemon balm-derived RA shows a high affinity for GABA_A_ receptors, a known pharmacological target for reducing insomnia symptoms [24,74]. As such, more sleep trials using sensitive research-grade actiwatches to measure sleep patterns [75] alongside magnetic resonance spectroscopy (MRS) to measure neural GABA [76] may reveal clearer sleep changes following lemon balm supplementation.

Studies exploring the effect of lemon balm on cognitive function were mainly carried out in healthy young adult populations and produced mixed results [29,45,46,47,48], with no consistent effects on cognitive domains, perhaps compounded by the lack of consistent dosages of lemon balm utilized in these studies. At face value, it is perhaps unsurprising that some studies report decrements in cognitive function following lemon balm administration, since the effects are sedative and calming as opposed to stimulating. Given the potential GABAergic mechanism of action, it would be of interest for future studies to explore whether there is a beneficial effect on tasks involving inhibitory processes compared to those that do not, as there are currently insufficient data to make such inferences. On the other hand, since cognitive performance can suffer under acute stress [77], lemon balm may help to buffer against feelings of stress and therefore stress-induced cognitive decline, as was tentatively suggested in one study [47]. Future studies should seek to further explore this potential in order to better understand where the balance between the calmative and cognitive effects lies. As only two studies explored the effect of lemon balm in older adults with mild dementia, it is not possible to determine whether lemon balm may be more effective in older adult populations compared to younger populations, given that one study reported improvements in ADAS-cog [64] and one did not [66]. Additionally, since there are no studies in healthy older adults, it is not possible to explore whether lemon balm may be more effective in healthy older adults experiencing natural age-related cognitive decline.

Lemon balm appears safe and tolerable given that vulnerable populations, including infants and hospitalized patients, reported no side effects and tolerated up to 20 days of daily 5000 mg intake [42,61,62,68]. In addition, reports of low drop-out rates over eight weeks of chronic lemon balm intake [55,61] and no evidence of safety problems in electrocardiogram and biochemistry examinations [52,55,63], are reassuring. Despite these promising findings, adverse effects arising from higher doses (>5000 mg) or in studies supplementing for longer than 56 days [55,61] are currently unclear. Nevertheless, evidence of a cardioprotective action of lemon balm outlined in a recent review [9] and a 90-day aqueous lemon balm oral toxicity rodent study (equivalency of 30.5–37.2 mg/kg body weight/day in humans) demonstrated a lack of genotoxic effects, supporting the safe use of food consumption above the age of two [78]. In addition, the lack of side effects following lemon balm intake may be a viable option to support GABA-targeted therapies [79] and AChE inhibitors, where adverse events of dizziness, vomiting, and diarrhoea are common [80].

While inconsistency in factors such as dose, format, and duration make the evidence more challenging to interpret, one of the key limitations to this field is how few studies report the phytochemical breakdown of the lemon balm intervention being used. Only six studies measured the individual phytochemical components of lemon balm, identifying 4.88 GA/g for phenolic content and 4.28 RU/g for flavonoid content in two studies [53,62] and various concentrations of hydroxycinnamates, essential oils, and triterpenes in others [49,63,65,67]. Three further studies provided the standardised content of active components such as RA [29,57,58], but with no evidence of phytochemical screening prior to study commencement. It is therefore important that future studies measure and report the phytochemical breakdown of the intervention used, alongside the use of more consistent dosing regimens, to further reveal the mechanistic action of lemon balm and identify whether individual components work independently or synergistically in vivo.

In general, across the lifespan, lemon balm supplementation in acute and chronic doses improved at least one psychological facet regardless of dose, duration, and/or psychological measure chosen. Future studies may benefit from performing a direct comparison of lemon balm benefits, for example, in young adults to older adults, to establish whether there is a target population for optimal lemon balm efficacy. Collectively, the available evidence supports lemon balm as a safe and efficacious option for a non-pharmacological choice among those wishing to self-administer as a potential treatment for resolving mild non-clinical psychological problems. Further robust RCTs with more controlled and standardized extracts with phytochemical profiling of the lemon balm extract are required to understand the efficacy of lemon balm as a treatment for a wide range of outcomes.
